# Could a chimeric condition be responsible for unexpected genetic syndromes? The role of the single nucleotide polymorphism‐array analysis

**DOI:** 10.1002/mgg3.546

**Published:** 2019-01-09

**Authors:** Roberta Bottega, Stefania Cappellani, Antonella Fabretto, Alessandro Mauro Spinelli, Giovanni Maria Severini, Michelangelo Aloisio, Michela Faleschini, Emmanouil Athanasakis, Irene Bruno, Flavio Faletra, Vanna Pecile

**Affiliations:** ^1^ Institute for Maternal and Child Health – IRCCS “Burlo Garofolo” Trieste Italy; ^2^ University of Udine Udine Italy

## Abstract

In this paper, is reported the identification of two chimeric patients, a rare finding if sexual abnormalities are absent. However, their chimeric condition is responsible at least for the Silver–Russell phenotype observed in one of the two patients. By single nucleotide polymorphism‐array analyses, it was possible to clearly define the mechanism responsible for this unusual finding, underlining the importance of this technique in bringing out the perhaps submerged world of chimeras.

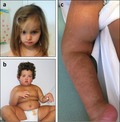

To the Editor

The term “chimerism” refers to the presence of two or more genetically distinct cell lineages originating from different zygotes in the same individual and can be easily distinguished from mosaicism by the extent of genotypic differences along the genome. Chimeras generally come to medical attention when they contain both male and female cells (46,XX/46,XY karyotype), causing disorders of sex development, or a discordance between external genitalia and chromosomal sex (Malan, Vekemans, & Turleau, 2006). In phenotypically normal individuals, chimerism may come to light only if there is a reason to perform genetic testing, that is, blood typing discrepancy (Drexler et al., 2005) or paternity testing (Ramsay et al., 2009). All the other chimeric individuals would not be detectable by standard cytogenetic technology, suggesting that this phenomenon might be underdiagnosed. About the origin of chimera individuals, four principal mechanisms have been identified as follows: (a) Tetragametic chimera (Green, Barton, Jenks, Pearson, & Yates, 1994); (b) Androgenetic chimera (Kaiser‐Rogers et al., 2006); (c) Fertilization of an ovum and a second polar body by two different spermatozoa and subsequent fusion of the two zygotes (Green et al., 1994); and (d) Parthenogenetic chimera (Giltay et al., 1998).

Herein, we present two patients characterized by a Silver–Russell‐ and Prader–Willi‐like phenotypes in which, despite a genetically normal karyotype detected in blood, a genomewide single nucleotide polymorphism (SNP) array analysis on DNA from skin biopsies highlighted a chimeric status.

We discuss SNP array as a technique able to identify chimerism also suggesting which one of the complex mechanisms underlying chimera formation could be responsible (Biesecker & Spinner, 2013). Subsequently, a custom next‐generation sequence (NGS) panel was used for chimerism quantification (Aloisio et al., 2016).

Based on the whole clinical picture (see Supporting Information Appendix S1; Figure 1), Patient 1 (female, P1) and Patient 2 (male, P2) underwent a classic and molecular (Human OmniExpress‐12 Bead Chip; Illumina Inc., San Diego, USA) karyotyping on peripheral blood in order to study microdeletion/duplication and UPD involved in Silver–Russell (SRS) and Prader–Willi (PWS) syndromes. This study was approved by an ethics committee and patients gave written informed consent to the investigation, according to the Declaration of Helsinki. No genetic alterations were detected. However, since the clinical presentation of patients includes also cutaneous hypopigmented striae following Blaschko's line along the trunk (P1) and in the limbs (P2; Figure 1c), a SNP array analysis on DNA from skin biopsy was performed in order to investigate a mosaic condition. Unexpectedly, the results showed a diffusely altered pattern of B allele frequencies (BAF) along all the autosomes that was consistent with the coexistence of two different genotypes with an altered ratio between the two haplotypes that could be explained by a chimeric status. Quantification of alleles frequency on skin fibroblasts, demonstrated the presence of two genomes with frequency of 30:70% in P1 (Figure 2a) and of 10:90%, in P2, respectively (Figure 2b). Although SNP array analysis shows a normal log ratio graph, the coexistence of two distinct genomes results in five different tracks (AAAA, AAAB, AABB, ABBB, BBBB) based on the number of allele combinations in autosomes. Moreover, as shown in Figure 2a, we could observe that the AABB splits into two tracks because of the 30:70% of coexisting genomes. This leads to a patchy BAF scattergram with four‐track interchanged with six‐track segments (Figure 2a). This arrangement, although present also in P2, is more difficult to identify because of the strong quantitative imbalance of the two coexisting genomes (10:90%).

**Figure 1:**
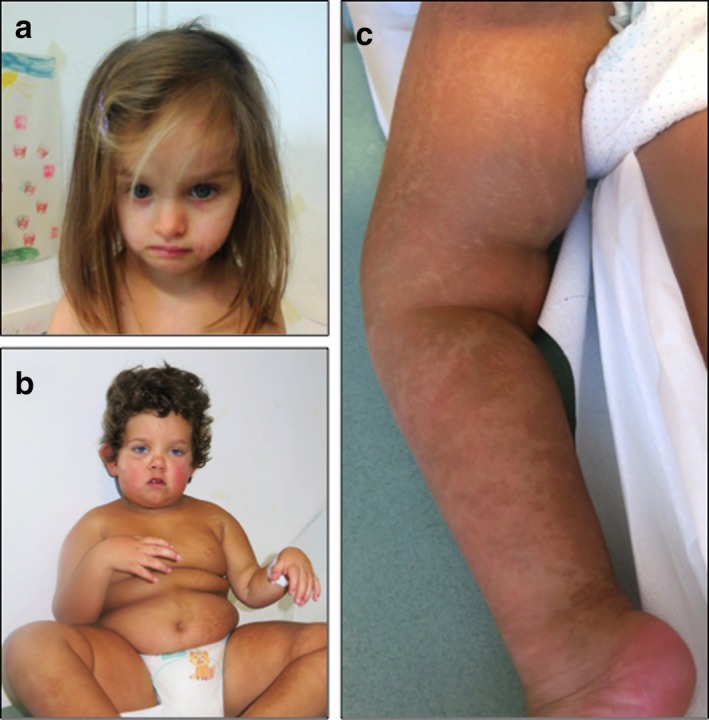
Clinical features. (a) Patient P1 showing asymmetrical face; (b) Patient P2 showing a hypotonic appearance, obesity, and slight dysmorphic features; (c) Patient P2 showing hyperchromic striae highlighting the coexistence of two distinct cell lines

**Figure 2:**
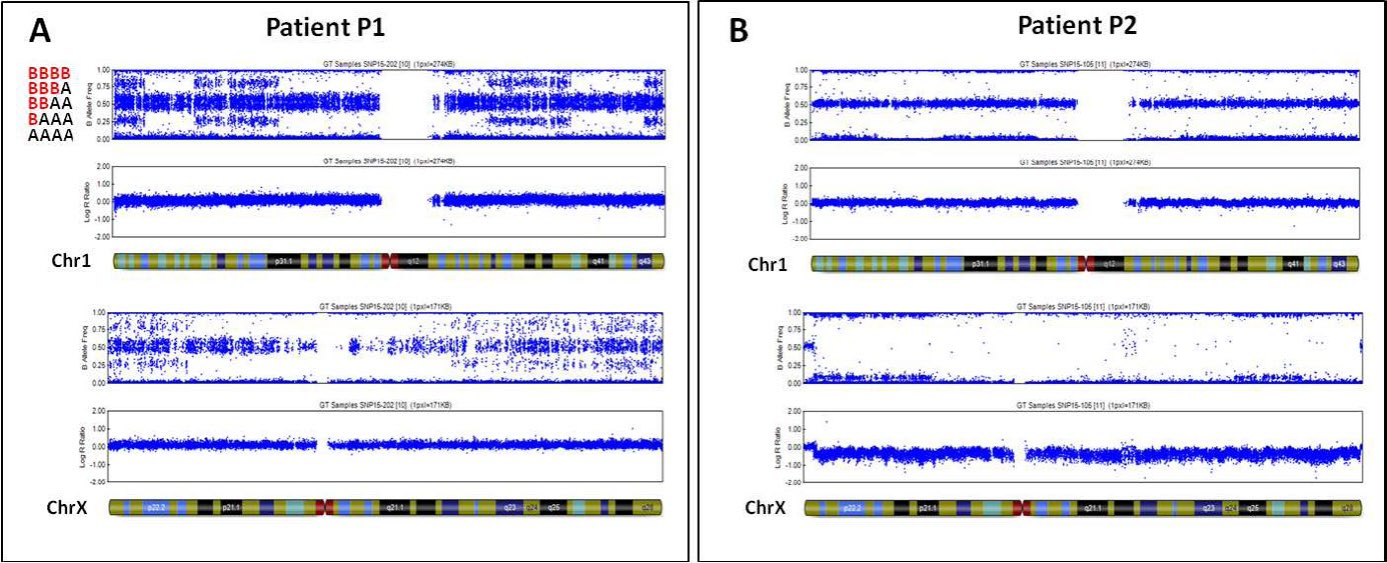
Scattergrams of B allele frequency (BAF) in single nucleotide polymorphism array analysis. (a) Patient P1’s scattergrams relative to chromosome 1 and X. (b) Patient P2’s scattergrams relative to chromosome 1 and X. All BAF graph show a patchy chromosomal pattern due to an altered alignments of different allele‐combination segments. In both patients, log ratio appears normal, according with sex

To better evaluate the percentage of chimerism in P1 and P2, we are taking advantage of a 44‐Amplicon Custom Chimerism panel based on Ion AmpliSeq technology as previously reported by Aloisio et al. (2016, identifying 36 SNPs shared with the SNP array chip.

Both NGS data from the blood and the skin DNA samples were collected thus determining the genotypic layout in both tissues. Considering as informative markers only SNPs found in homozygous state (i.e., AA) in the blood sample and in chimeric state (i.e., AG or GG) in the skin sample, it was possible to determine the chimeric allele and the two possible genotypes in the skin. So two distinct chimerism values were possible (homozygous or heterozygous state): in P1, 16% or 32% and in P2 3.5% or 7%, respectively (Table 1); however, the NGS analysis can not discriminate the right value. For these reasons, the comparison between the SNP array and NGS approach suggests that although the quantification of the degree of chimerism is more accurate using a NGS approach, the SNP array is able not only to provide values that do not significantly deviate from those obtained by NGS, but also to discriminate which of the two possible percentages reported by the NGS output is the most likely.

**Table 1:** Informative single nucleotide polymorphisms e chimerism percentage calculated by next‐generation sequence approach

	Markers		Chimeric allele count (%)	Predicted chimerism %, based on Skin genotype	
	rs SNP	Blood genotype		Skin genotype	Skin genotype
P1	rs1561570	TT	C (15%)	TC (30%)	CC (15%)
	rs2077163	TT	C (16%)	TC (32%)	CC (16%)
	rs10033900	TT	C (17%)	TC (34%)	CC (17%)
	Average chimerism value:			32%	16%
P2	rs2297313	AA	G (4%)	AG (8%)	GG (4%)
	rs10489266	AA	G (3%)	AG (6%)	GG (3%)
	rs8128316	CC	T (3%)	CT (6%)	TT (3%)
	rs744166	GG	A (4%)	GA (8%)	AA (4%)
	rs10143250	TT	C (4%)	TC (8%)	CC (4%)
	rs132985	TT	C (3%)	CT (6%)	CC (3%)
	Average chimerism value:			7.0%	3.5%

A further advantage in using the SNP array approach is represented by the possibility to determine the parental origin of the haploid genomes, which contributed to the chimera formation, by comparing patients and parents’ SNP array results. This analysis demonstrated that segments observed in the BAF of both P1 and P2 are clearly defined by contribution of two different maternal alleles (AA or BB, determining the three allele‐combination segments of AAAA, AABB, or BBBB), that joining with the paternal contribution led to the five different allele‐combination segments of AAAA, AAAB, AABB (divided in our case as explained above), ABBB, or BBBB. The changing points between three and five allele‐combination segments in autosomes indicate the crossover points.

In patient P1, a female, BAF configuration through the X‐chromosome shows the same pattern observed in autosomes indicating two different maternal contributions (Figure 2a) otherwise, in patient P2, log ratio of the X‐chromosome appear normal, according to his gender (male) while BAF configuration shows four different tracks due to the two maternal contributions in 90:10%, respectively (Figure 2b).

As a final task, the use of SNP array has allowed us to understand which mechanism has presumably been the cause of the chimera formation for both patients. In fact, in our cases, the androgenetic and the parthenogenetic chimera were excluded because their output were incompatible with our SNP array’ results as in our BAF two different maternal genomes have been showed (Giltay et al., 1998).

Chimerism found in P1 could be explained by a fertilization of one nucleus by a normal spermatozoa and endoreduplication of the polar body (Figure 3). Conversely, the pathogenetic mechanism behind the P2 is compatible with a tetragametic chimera because the SNP analysis detected five haplotypes that could be explained by two oocytes fertilized by two spermatozoa or fertilization of an ovum and a second polar body by two different spermatozoa (Figure 3). It is really challenging to discriminate these hypotheses because of the high difference between the percentages of the genomes.

**Figure 3:**
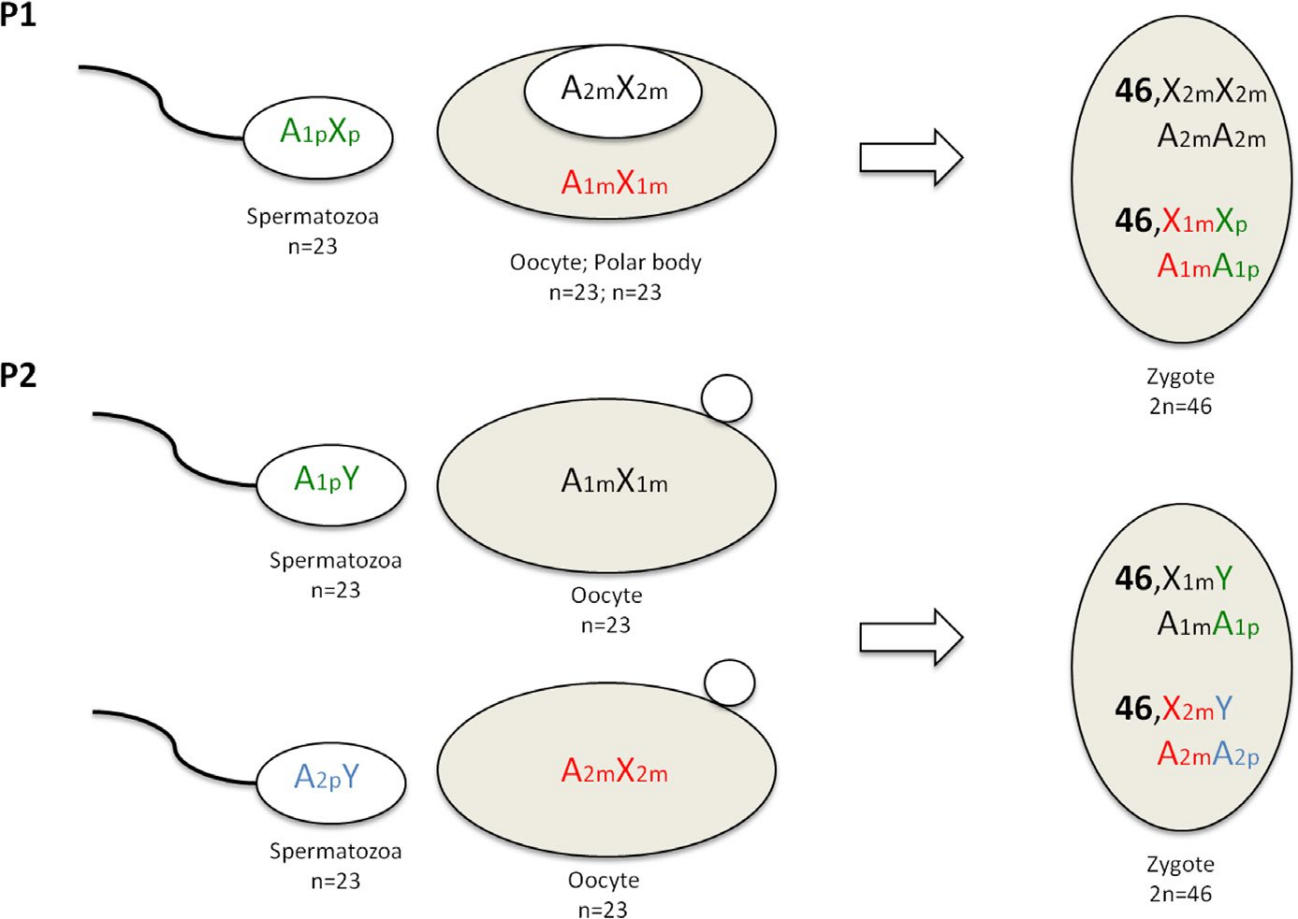
Proposed mechanisms to explain chimera in our patients. Fertilization of the oocyte by a spermatozoa and endoreduplication of the polar body in P1. Tetragametic chimera in P2

In light of chimeric determination in patients, it is possible to make some considerations on the patients' phenotype. Regarding the Silver–Russell‐like phenotype in the female patient (P1), it could be explained by the uniparental isodisomy in the endoreduplicated polar body genome (a mechanism first proposed by Yamazawa et al. (2010. Otherwise, the diagnosis of Prader–Willi‐like syndrome for patient P2 could not be directly explained by its chimeric status. Since a MS‐MLPA in the 15q11 region detects a normal pattern of methylation and no deletion, we suggest in P2 a PWS‐like phenotype for which we cannot exclude the chimeric involvement.

In conclusion, many cases of whole‐body chimerism are reported in literature; unfortunately most of them present insufficient molecular information allowing to clearly define the mechanism involved in the formation of individual cases. Nonetheless, several cases of proven tetragametic and parthenogenetic chimeras have been described based initially on chromosome heteromorphisms, and more recently on genotyping (Russo et al., 2016; Yamazawa et al, 2010).

Regarding our two patients, the observation of a chimeric state was an unexpected finding that could probably explain the syndromic status at least in P1 while in P2 a genetic mutation in one of the two coexisting genomes could be the causal event.

To assume chimerism, a priori in the absence of substantial clinical abnormalities is very difficult also because, as demonstrated in our cases, not all the tissues express both genomes, probably leading to an underestimation of this phenomenon. From this perspective, the use of genomewide SNP arrays enables simultaneous evaluation of genomic dosage allowing insights into the mechanisms by which these abnormalities occur. For these reasons, we suggest that the routine use of genotyping SNP arrays analysis for the identification of “hidden” chimerism in patients with few clinical clues.

## CONFLICT OF INTEREST

The authors declare no conflict of interest.
