# Supplementary material for: Could a chimeric condition be responsible for unexpected genetic syndromes? The role of the single nucleotide polymorphism‐array analysis
Source: Mol Genet Genomic Med. 2019 Jan 9;7(3):e546. doi: 10.1002/mgg3.546 (PMC6418439; doi:10.1002/mgg3.546)
Supplement: Supplementary file 1 [file MGG3-7-na-s001.docx]

**Supplementary data**

*Patient clinical features*

Patient P1 (Figure 1A). The patient is a full term female infant born small for gestational age after a pregnancy characterized by intrauterine growth retardation. The family history was negative for genetic diseases and the parents were not related. She was born at term and her anthropometric values were: W 2010 gr., L 45 cm and HC 31,8 cm (all below the 3rd percentile). The Apgar score was 7 and 8 at 1 and 5 minutes. At two years of age, she presented with hypotonia, hemihypertrophy, triangular facies, frontal bossing, micrognatia and bilateral clinodactyly. Moreover, she had growth delay characterized by height and weight lower than 3° percentile, but with normal head circumference. The motor milestones were reached timely, although she showed a language delay. Full scale IQ was at the lower end of the normal range. Because of her facial and body asymmetry she was treated for the stomatological and orthopedic problems. When the baby was 6 years old she presented with hypopigmented striae following Blaschko’s lines in the limbs.

Patient P2 (Figure 1B,C). P2 was born to healthy unrelated Italian parents and he has a healthy older sister. His pregnancy was characterized by onset of IUGR and oligohydramnios during the second trimester. The mother performed chorionic villus sample, which revealed a normal 46,XY karyotype. He was born at 32 gestational weeks and stayed in NICU for several weeks because of low birth weight (1100 gr) and respiratory difficulties related to prematurity. No suction or swallowing abnormalities were reported. After discharge the patient was followed for several developmental difficulties both on motor and cognitive side. He reached the sitting position at 18 months and walked with support at 24 months. Electromyography revealed no abnormalities. On the cognitive side there is also e cognitive impairment and the boy presents a severe speech delay with absent words at 2 yrs and half. Audiometry revealed mild conductive hearing loss. Growth was below the 3° centile for height and weight until the first year of life; afterwards height followed the lower centile while weight increased dramatically exceeding the 97° centile. At last visit, (36 months) his weight was 18kg (>97°centile), height was 89cm (10° centile), and OFC was 51cm (90° centile). More peculiar signs are two sacral dimples and bilateral cryptorchydism. More exams are performed as abdominal US and cardiological examination which indicate only mild hepatomegaly due to steatosis. Blaschkoid dyspigmentation was evident along the trunk, especially on the left side.
